# Predictors of low prevalence of latent tuberculosis infection among Egyptian health care workers at intensive care and bronchoscopy units

**DOI:** 10.3205/dgkh000282

**Published:** 2016-10-12

**Authors:** Enas Mamdouh Hefzy, Ahmed Ashraf Wegdan, Radwa Ahmed Elhefny, Samar Hassan Nasser

**Affiliations:** 1 Department of Medical Microbiology and Immunology, Faculty of Medicine, Fayoum University, Fayoum, Egypt; 2 Department of Chest Diseases, Faculty of Medicine, Fayoum University, Fayoum, Egypt; 3 Fayoum General Hospital, Ministry of Health, Fayoum, Egypt

**Keywords:** health care workers, infection control, latent tuberculosis, QuantiFERON-TB Gold test, tuberculin skin test

## Abstract

**Aim:** Latent tuberculosis infections (LTBI) contain a significant reservoir for future epidemics. Screening of health care workers (HCWs) in a high-risk tuberculosis (TB) environment is an important strategy in TB control. The study aimed to assess the prevalence of LTBI among high risk Egyptian HCWs and to assess infection associated risk factors.

**Methods:** Fifty-two HCWs who work at intensive care unit (ICU), bronchoscopy unit, and chest diseases department were tested for LTBI using both tuberculin skin test (TST) and Quantiferon TB Gold in-tube test (QFT). Risk factors for infection, knowledge of HCWs towards different aspects of TB infection and agreement between TST and QFT were also evaluated.

**Results:** Prevalence of LTBI in this study was 13.5% by QFT and TST. It was 13.6% by TST alone and 10.3% by QFT alone. There was good concordance between both tests (Kappa=0.713). There was a statistically significant association between prevalence of LTBI and age of staff ≥30 yr (p=0.002), period of working experience (p=0.006) and working at the Bronchoscopy Unit (p=0.001). The total knowledge of HCWs towards different aspects of TB infection was generally good.

**Conclusion:** Although the participants in the current study were among high risk HCWs, the prevalence of LTBI was low. Bacille Calmette-Guerin (BCG) vaccination, young age, short employment duration, good knowledge and a good infection control were the predictors of low risk of contracting TB at our hospitals. The risk of TB infection in resource-limited countries can be reduced with simple continuous educational and administrative infection control programmes.

## Zusammenfassung

**Zielsetzung:** Eine latente Tuberkuloseinfektion (LTBI) ist ein Reservoir für Epidemien. Daher ist das Screening von medizinischem Personal (HCWs) in einem Hochrisikobereich eine wichtige Strategie zur Kontrolle der Tuberkulose (TB). In der Untersuchung sollten die Prävalenz von LTBI bei ägyptischem medizinischem Personal und mit der Infektion assoziierte Risikofaktoren bestimmt werden.

**Methode:** 52 HCWs, die auf einer Intensivtherapieeinheit (ITS), einer Bronchoskopieeinheit und einer Abteilung für Thoraxerkrankungen tätig waren, wurden auf das Vorkommen einer LTBI mittels Tuberkulin-Hauttest (THT) und QuantiFERON-TB Gold (QFT) untersucht. Ferner wurden Risikofaktoren für eine Infektion, Kenntnisse der HCWs zu verschiedenen Aspekten der TB-Infektion und die Übereinstimmung zwischen THT und QFT evaluiert.

**Ergebnisse:** Die Prävalenz von LTBI betrug 13,5% mittels QFT und TST (13,6% mittels TST allein und 10,3% mittels QFT allein). Damit war eine gute Konkordanz zwischen beiden Tests gegeben (Kappa=0.713). Es war eine statistisch signifikante Assoziation zwischen der Prävalenz von LTBI und Alter der HCWs ≥30 Jahre (p=0.002), Zeitdauer der Tätigkeit (p=0.006) und Tätigkeit in der Bronchoskopieeinheit (p=0.001) nachweisbar. Die Kenntnisse der HCWs zu verschiedenen Aspekten der TB-Infektion waren im Allgemeinen gut.

**Schlussfolgerung:** Obwohl die Teilnehmer der Studie in Hochrisikobereichen tätig waren, war die Prävalenz der LTBI niedrig. Bacillus Calmette-Guérin (BCG)-Impfung, junges Alter, kurze Zeitdauer der Berufstätigkeit, gute Kenntnisse und eine gute Infektionskontrolle erwiesen sich als Prädiktoren für ein geringes Risiko zum Erwerb einer LTBI. Das Risiko einer TB-Infektion kann in Ländern mit begrenzten Ressourcen durch einfache kontinuierliche Erziehung und Infektionskontrollprogramme reduziert werden.

## Introduction

Tuberculosis (TB) is one of the common and, in many cases, lethal infectious disease in the world. In several studies, health care workers (HCWs) appear as a subpopulation with a higher risk of a TB infection than the remaining population without occupational exposure [1]. Nosocomial transmission of *Mycobacterium tuberculosis* (*M. tuberculosis*) from patients to HCWs has been identified for many years; the risk of transmission is the greatest in facilities with a high burden of infectious TB cases [2].

Currently, two methods are available for the evaluation of latent tuberculosis infection (LTBI): a tuberculin skin test (TST) and interferon-gamma release assays (IGRAs) [1]. TST depends on delayed hypersensitivity reaction to purified protein derivative (PPD) of *Mycobacterium bovis*. TST sensitivity ranges from 75%–90% with limited specificity, especially when exposure to *M. tuberculosis* is common, due to cross-reactivity of PPD with the Bacille Calmette-Guérin (BCG) vaccine and non-tuberculous mycobacteria (NTM). The inter-observer variability, boosting phenomenon and the need for a reading visit besides false-negative results in older persons, young persons and immune-compromised patients are other limitations of the TST use [3]. 

A hallmark of the immune reaction to *M. tuberculosis* infection is the interferon (IFN)-γ release by CD4 cells. Measurement of this response in blood samples to culture filtrate protein 10 (CFP10) and early-secreted antigen 6 (ESAT6), highly specific MTB antigens, is the basis of IGRAs test. These antigens are not present in BCG or NTM, thus IGRAs are more specific than TST. Other important advantages of the IGRAs include objective output and a single patient visit [4].

Latent TB infections contain a significant reservoir for future epidemics. Screening of HCWs working in a high-risk TB environment by chest X-rays and TST, LTBI targeted treatment and chemoprophylaxis administration in high-risk groups are important strategies in TB control [5]. TB transmission in health-care facilities can be prohibited with implementation of effective infection control measures [6]. Control measures of TB infection have been highly spotted recently by data of the development and spread of extensively drug resistant (XDR)-TB which is linked with mortality and morbidity [7].

Incidence of TB in Egypt was last measured as 15 (per 100,000 people) in 2014, according to the World Health Organization (WHO) [8] but little is known about the prevalence of LTBI among Egyptian HCWs. The current study aimed to assess the prevalence of LTBI among HCWs at Fayoum University Hospital (FUH) who are at high risk using both TST and Quantiferon TB Gold in-tube test (QFT) which is an IGRA test. Assessment of risk factors for infection and agreement between TST and QFT were also evaluated.

## Methods

### Study participants

This cross-sectional study was conducted from August 2015 to January 2016 at FUH. The centre is a 500-bed referral hospital that serves Fayoum Governorate which is a rural governorate with about three millions population. Annual prevalence of active TB has been reported as 200 cases per year. About 300 brochoscopes are performed yearly at FUH. The study aimed to assess the prevalence of LTBI among HCWs who are at high risk, based on exposure to patients with active tuberculosis. They were from Intensive Care Unit (ICU), Bronchoscopy Unit, and Chest Diseases Department. All participants gave their written informed consent before their inclusion in the study. The study protocol was approved by the Research Ethical Committee at Faculty of Medicine, Fayoum University.

### Data collection

Each participant included into the study has completed a self-administered questionnaire containing information about: possible risk factors (sociodemographic, occupation history, smoking, BCG vaccination, co-morbid diseases, history of previous TB, and history of contact or living with TB patient) and knowledge about LTBI among physicians, nurses and housekeepers at FUH. This second part contained questions with 28 items covering knowledge about TB transmission, LTBI, TB vaccination and treatment, TST and prevention of TB transmission (personal protective equipment and hand washing). This questionnaire was modified from that developed by Montagna et al. [9]. Degree of knowledge was ascertained by means of “yes”, “no” or “don't know” questions on each item being evaluated.

The questionnaire was translated into Arabic to be easily understood and answered. It took approximately 15 minutes to complete it. Before administration of the questionnaire, the purpose of the study was explained to each respondent and confidentiality of the information was assured. Each statement of knowledge was measured on three points; a right answer was scored two points, an “I do not know” answer was scored one point, and a wrong answer was scored zero points with a maximum total score of 56. In this study, a diagnosis of LTBI was made if the respondent was tested positive by TST and/or QFT.

### Quantiferon TB Gold in-tube test 

Quantiferon TB Gold in-tube test (QFT) (Cellestis/Qiagen) was performed according to the manufacturer’s instructions. Briefly, one ml of whole blood was sampled in each of the three QFT tubes containing TB specific antigen (CFP-10, ESAT-6 and TB7.7), mitogen antigen (positive control) and no antigen (negative control). Tubes were incubated for 16 to 24 hr at 37°C then plasma was harvested from each tube. An enzyme-linked immunosorbent assay (ELISA) reader was used to measure the IFN-γ concentrations (IU/ml) in plasma and this value was calculated by the ‘QFT-TB-analysis software’. A value ≥0.35 IU/ml (TB antigens minus negative control) was considered as a positive test.

### Tuberculin skin test

TST was done after blood for QFT has been withdrawn to avoid the possibility of reaction boosting. The test was administered using the Mantoux method i.e. intradermal injection of 0.1 ml of PPD containing 5TU (Tuber test, vacsera, Egypt) and read after 48–72 h. Induration of ≥10 mm was considered a positive result for HCWs. 

The study participants with a positive QFT or TST were followed according to clinical practice with clinical and radiological examination to rule out active TB. 

### Data analysis

All HCWs who agreed to participate were included for the analysis. The collected data was organized, tabulated and statistically analyzed using SPSS software statistical computer package version 18 (SPSS Inc, USA). For quantitative data, the mean and standard deviation (SD) were calculated. Independent t-test or one way ANOVA were used, when appropriate, to test the differences between several study variables as regards mean values of knowledge score. For qualitative data the number and percentage were calculated. Chi squared test (χ^2^) was used as a test of significance. Agreement between the two tests, TST and QFT, and Kappa values were calculated. The general significance was adopted at P≤0.05. 

## Results

### Socio-demographic characteristics of study population 

This was a cross-sectional study included 52 HCWs who are at high risk for exposure to TB patients and therefore infection. The age of participating HCWs ranged between 19 and 50 years old with the mean age was 28.8 ± 7.5, 24 (46.2%) of participants were males and 28 (53.8%) were females. Their job distribution was as follows; 31 (59.6%) were nurses, 13 (25.0%) were housekeepers and 8 (15.4%) were physicians. Experience of HCWs in working at health care facility ranged from 0.5 year to 20 years with mean ± SD of 4.9 ± 4.7. All respondents were BCG vaccinated as assessed by history and/or vaccination scar. Seventeen HCWs (32.7%) reported a suspected exposure to TB case or infected specimen and one participant (1.9%) to a diseased relative. Socio-demographic and occupational data of participants is shown in Table 1 (Tab. 1). 

### Knowledge of studied participants as regards latent TB 

Table 2 (Tab. 2) demonstrates the responses and scores of knowledge of HCWs towards different aspects of TB infection. The total knowledge of the study group regarding TB was generally good with a total score of 44.2 ± 5.2 out of 52. Regarding LTBI, the majority of participants have mentioned the correct answers with a percentage over 50 for all items except for two questions; TB is mostly asymptomatic and there are many methods for diagnosing LTBI where less than half of HCWs mentioned the correct answer. 

For questions about treatment and specific protection against TB, participants mentioned the correct answers with a percentage over 50 for three questions; treatment of TB need long duration, treatment of TB is complicated and there is a vaccine for TB. On the other hand, less than half of HCWs gave correct answers for the other questions (Table 2 (Tab. 2)). According to TST, the majority of participants could not mention the correct answers for all items except one question; negative TST results mean no infection where more than half of HCWs identified the correct answer. Knowledge of the HCWs as regards measures of prevention was high. The majority of participants could mention the correct answers with a percentage over 80 for almost all items (Table 2 (Tab. 2)). 

Table 3 (Tab. 3) specifies that there was a statistically significant difference between total knowledge score among different age groups, working experience and working activities, P=0.004, 0.024 and <0.0001, respectively. Poor knowledge score was found among older age (≥30 years old), working experience ≥10 years and housekeepers.

### Prevalence and risk factors of LTBI among study participants

Overall prevalence rate of LTBI among the studied HCWs, detected by TST and/or QFT, was 13.5% (7/52). By TST alone, prevalence was 13.6% (6/44), while by QFT alone it was found to be 10.3% (4/39), as shown in Table 4 (Tab. 4). Thirty one of participants agreed to be tested by both tests with 26 (83.9%) were negative by both tests, three (9.7%) were positive in both tests, one was positive by TST alone and one was positive by QFT alone. There was good concordance between both tests (Kappa=0.713).

Out of the study participants, 39 agreed to be tested for LTBI with QFT. Of the respondents, four participants gave positive results but only three of them were positive when tested with TST. Of the participants with negative results by QFT (=35), 27 were screened for LTBI by TST and one participant only gave positive results.

Table 5 (Tab. 5) reveals a statistically significant association between prevalence of LTBI and age of staff ≥30 yr (p=0.002). There was a statistically significant association between prevalence of LTBI and period of working experience (p=0.006) and working at the Bronchscopy Unit (p=0.001). Although, LTBI was more common in housekeepers (30.8 %) than nurses (9.7%) no statistically significant association was found (p>0.05). Other socio-demographic parameters were not significantly associated with prevalence of LTBI (p>0.05). Table 5 (Tab. 5) demonstrates prevalence of LTBI among different socio-demographic and occupational groups of participants.

Table 6 (Tab. 6) specifies that there was no statistically significant difference between mean total knowledge score of the infected and the not infected groups. As regards, subtotal knowledge scores, there was a statistically significant difference between the infected and the not infected groups in knowledge about LTBI, (mean ± SD was 6.7 ± 1.8 for who has vs. 8.6 ± 2.0 for who doesn’t have LTBI, p=0.025).

## Discussion

In low and middle income countries with high and intermediate TB burden, TB is a prominent occupational hazard among HCWs. In this cross-sectional study we aimed to assess the prevalence of LTBI among BCG vaccinated Egyptian HCWs from ICU, Bronchoscopy unit, and Chest Diseases Department. Those locations were identified as high-risk locations for LTBI within facilities [10], [11]. Prevalence of LTBI in this study was 13.5% by QFT and TST. It was 13.6% by TST alone and 10.3% by QFT alone. However, it is difficult to decide if the prevalence of LTBI among HCWs is significantly higher or lower than the community as data on concurrent LTBI prevalence in the community is not available to the authors. 

This low prevalence in Egypt, an intermediate TB burden country, was in concordance with prevalence of LTBI among HCWs in Malaysia, also an intermediate TB burden country, where overall prevalence was 10.6% [12]. The prevalence in Germany and Japan was 9.9% [13], [14]. Even though the prevalence is close to our findings, it should be prominent that Germany and Japan are, according to TB-burden, low-burden countries. Definitely, our findings are higher than those obtained from studies from countries with low TB burden like Denmark and Norway with prevalence of 1% and 3.4% respectively [15], [16]. 

The prevalence of LTBI in this study was relatively low in comparison with other low or middle income African countries where researchers found rates of LTBI among HCWs of 33% in South Africa, 57% in Uganda up to 79% in Côte d'Ivoire [17], [18], [19] with estimated pooled prevalence of 54% (95% CI 53–55) [2]. These higher values could be attributed to high prevalence of HIV infection in these countries or use of TST only for diagnosing LTBI which accounts for this higher prevalence with false positive reactions that could be attributed to NTM infections or BCG vaccination [20]. 

Although the participants in the current study were among high risk HCWs, the prevalence of LTBI was low. Concurrent data was obtained from Malaysia and Taiwan where no increased risk of LTBI among HCWs in direct contact with TB patients was reported [12], [21]. Also, the inclusion of young aged HCWs with short duration of occupation could be another explanation.

In this, BCG-vaccinated, studied population only 31 respondents accepted to be investigated by both TST and QFT with 93.5% agreement between both tests, Kappa=0.713. All TST positive respondents had readings ≥15 mm, this can explain the agreement between both tests. These findings parallel those obtained by Rafiza et al., where concordance between TST and QFT at cut-off values of 15 mm was 82.1% [12]. In agreement with our results, an earlier study in India reported high concordance between the two tests although majority of the respondents were BCG vaccinated [22]. A study in Germany, where BCG vaccination is not mandatory, Nienhaus et al., found good concordance among those who did not receive BCG vaccination and poor agreement among those who received vaccination [23]. This poor agreement between both tests has been attributed to BCG vaccination, NTM exposure and the cumulative occupational plus non-occupational exposure to *M. tuberculosis* [21].

Increasing age, more exposure to TB patients and duration of employment in the health care setting (denoting longer accumulative exposure), were risk factors for acquiring infection, which supports nosocomial transmission. Researchers reported about one time increase in the prevalence of LTBI in HCWs with each additional year of age [19], [10], and 1.5–2.4 times increase with employment duration of more than one year [24], [10] and 3-fold higher risk with more than 10 years of employment [22]. By univariate analysis, findings of this study support this data as there was a significant association between LTBI and age ≥30 years (p=0.002) and period of working experience ≥10 years (p=0.006). Similar results were reported in earlier studies [12], [19], [21], [25]. Another study from Rwanda found that within health care facilities, risk of infection did not differ significantly between occupations and work locations and reported that increased infection with TB within health care settings is affected by duration of employment in health facilities, regardless of department type or occupation [26]. In contrast, Gran et al. did not find any relation between age and the QFT results [27]. Also, Franchi et al. found that TST conversion was not associated with duration of time in contact with the infectious TB patient, but related to unprotected brief exposure to a highly infectious person in a closed and poorly ventilated confined spaces [28]. 

As both women and men in our study shared the same workplace medium, this may have reduced the significant association between sex and LTBI. In contrast, being male was significantly associated with LTBI in a study by Rafiza et al. [12]. Although LTBI was more common in housekeepers (30.8 %) followed by nurses (9.7%) but there was no statistically significant association was found (p>0.05). Sherman et al., also reported higher prevalence of LTBI among housekeepers [11]. A previous study, reported a lower prevalence in nurses, compared to other HCWs [29]. In contrast, it was reported in several studies that the prevalence of LTBI in nurses was higher than that in other HCWs [10], [12], [30]. 

The total knowledge of study group regarding TB was generally good which can explain the generally low prevalence of LTBI among the studied group. But there was a statistical significant difference between the infected and the not infected groups in knowledge about LTBI, (mean ± SD was 6.7 ± 1.8 for who have vs. 8.6 ± 2.0 for who do not have LTBI, p=0.025). In a case-control study by Jelip et al., HCWs with TB infection were about six times (95% CI 0.76–46.4) more likely to have poor knowledge about transmission of TB, and about four times (95% CI 0.95 to 19.8) less aware about the need for respiratory protection [31]. A 2.6-fold (95% CI 1.06 to 6.64) increase in risk of TB disease was associated with failure of personal protection among HCWs [32]. In the current work, poor knowledge score was found among older age (≥30 years old), working experience ≥10 years and housekeepers (P=0.004, 0.024 and <0.0001, respectively), which are the same risk factors associated with prevalence of LTBI. This indirectly reflects the positive correlation between poor knowledge and prevalence of LTBI.

## Conclusion

Based on our results we can conclude that young age, short employment duration, good knowledge and good infection control are the predictors of low risk of contracting TB at our hospital. Taken together, the risk of TB infection in resource-limited countries can be reduced with simple continuous educational and administrative controls, development of suitable infection control, surveillance programs and BCG vaccination. But larger studies are needed to evaluate this neglected chronic problem of health care associated TB in low income countries especially with the extensive drug resistant tuberculosis recent emergence.

## Notes

### Competing interests

The authors declare that they have no competing interests for this work.

### Funding

No funds were received for this work. 

## Figures and Tables

**Table 1:**
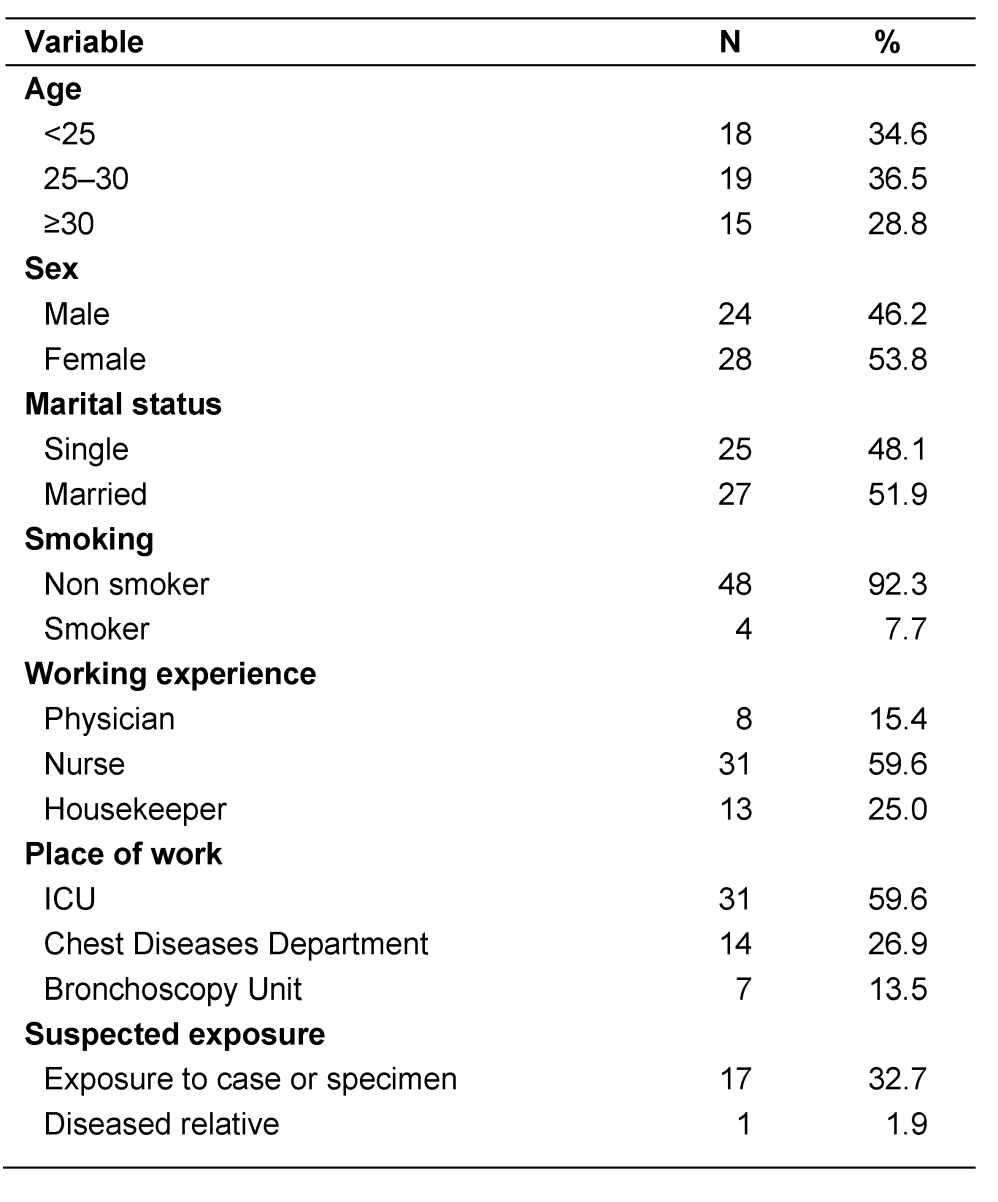
Socio-demographic characteristics of the study participants

**Table 2:**
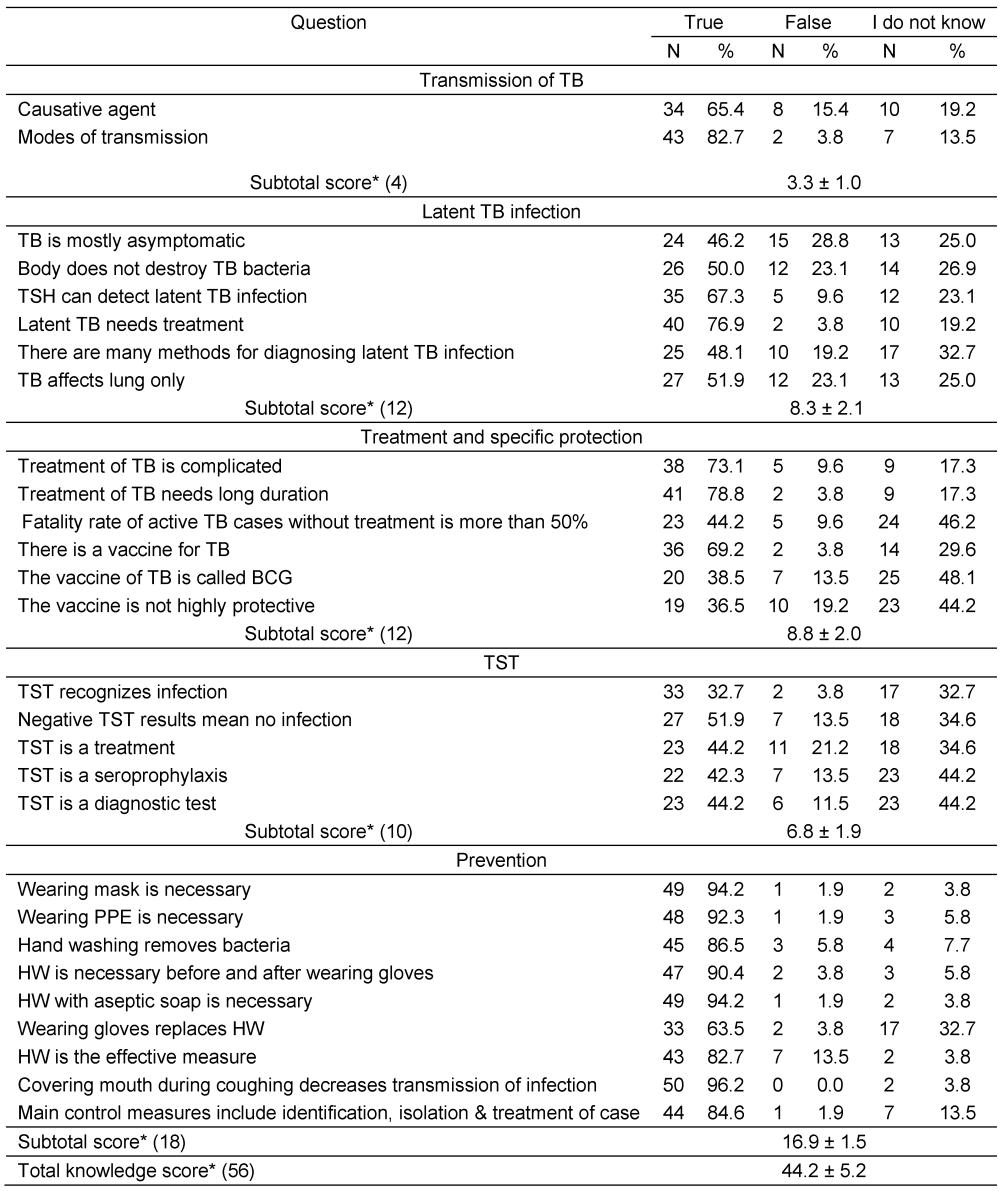
Knowledge of study participants

**Table 3:**
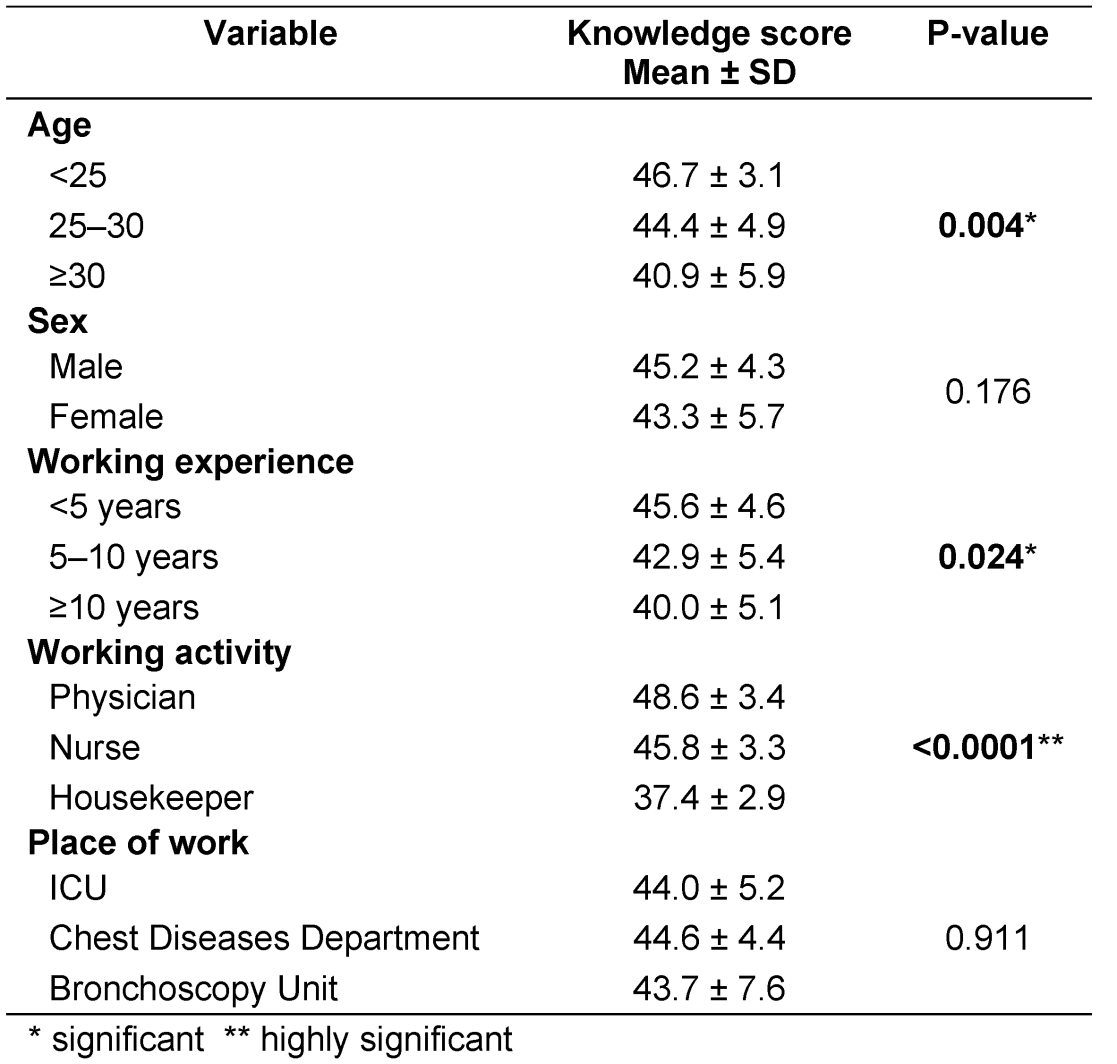
Differences between characteristics of participants as regards knowledge score

**Table 4:**
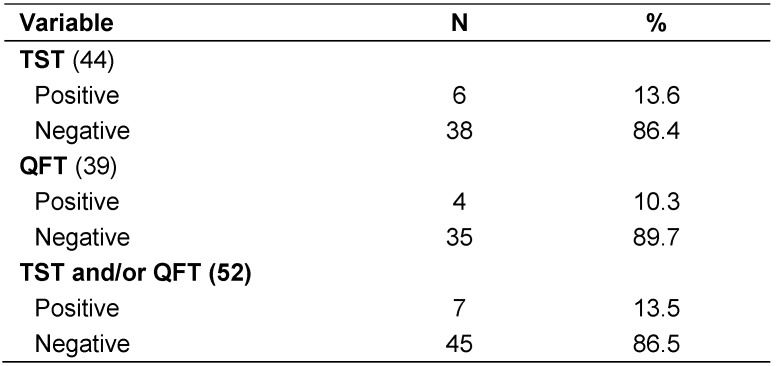
Prevalence of latent TB infection

**Table 5:**
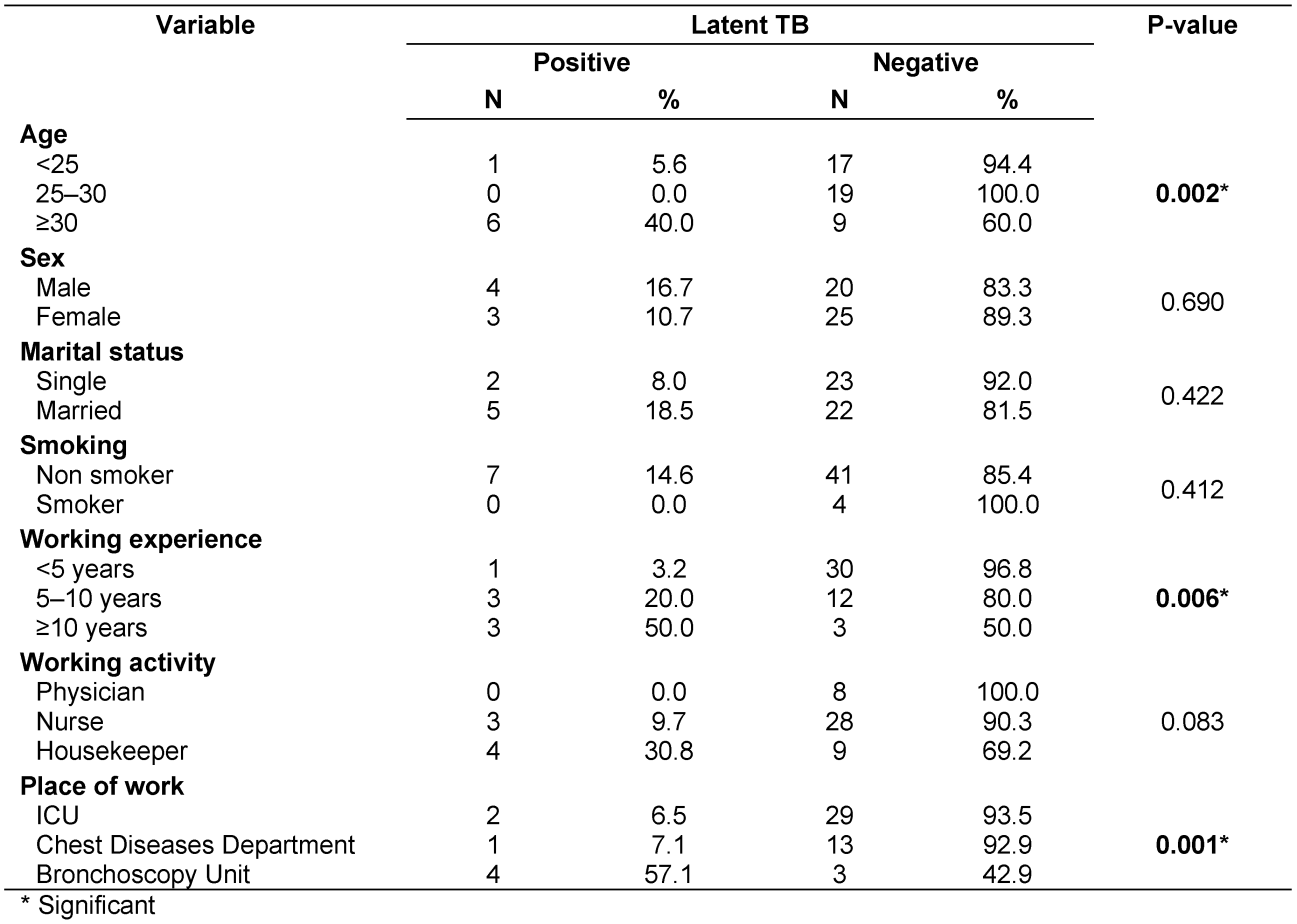
Relation between latent TB and socio-demographic factors

**Table 6:**
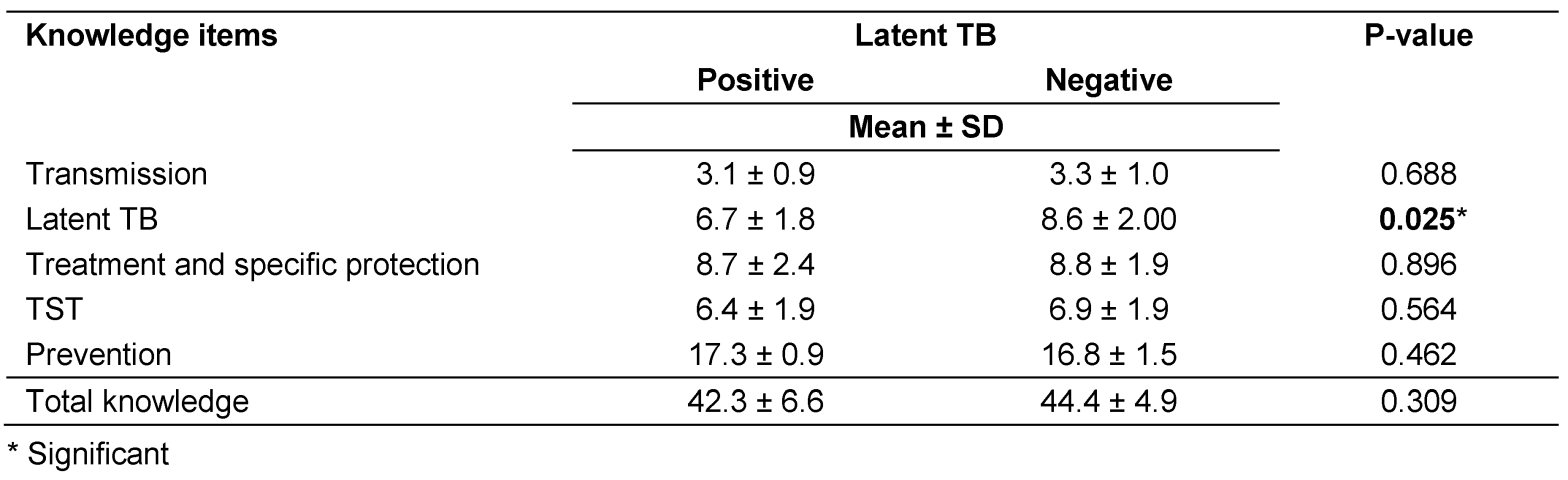
Relation of latent TB to knowledge of participants
